# Comparative Functional and Morphological Data of Different IOL Dislocation Treatment Methods

**DOI:** 10.3390/jcm14051462

**Published:** 2025-02-21

**Authors:** Renata Vaiciuliene, Ugne Rumelaitiene, Martynas Speckauskas, Vytautas Jasinskas

**Affiliations:** 1 Department of Ophthalmology, Faculty of Medicine, Medical Academy, Lithuanian University of Health Sciences, 44037 Kaunas, Lithuania; 2 Department of Ophthalmology, Hospital of Lithuanian University of Health Sciences Kaunas Klinikos, 50161 Kaunas, Lithuania

**Keywords:** late spontaneous IOL–capsular bag complex dislocations, IOL fixation to the iris, anterior chamber IOL

## Abstract

**Background:** This study compared the visual and morphological outcomes between iris fixation and anterior chamber intraocular lens (ACIOL) implantation for late spontaneously dislocated intraocular lens (IOL)–capsular bag complexes in a tertiary reference center in Lithuania. **Methods:** A prospective observational study was conducted between 2017 and 2019 involving 80 patients (83 eyes) with late spontaneous IOL–capsular bag dislocation. Patients underwent repositioning and fixation of the dislocated IOL to the iris (IF group) or IOL exchange with an ACIOL implant (ACIOL group). Pre- and postoperative assessments included best-corrected distance visual acuity (BCDVA), intraocular pressure (IOP), corneal endothelial cell density (ECD) and macular thickness (evaluating whether cystoid macular edema (CME) had occurred). **Results:** Both groups showed a significant improvement in BCDVA, with a more remarkable improvement in the IF group (median: 0.1 logMAR) than in the ACIOL group (median: 0.3 logMAR), *p* = 0.001. Corneal astigmatism increased significantly in the ACIOL group (*p* < 0.001) but remained stable in the IF group. IOP management outcomes were better in the IF group as fewer eyes required additional glaucoma treatment. ECD decreased in both groups, but the decrease was significantly greater in the ACIOL group (*p* < 0.001). Postoperative CME occurred in 4.4% of IF eyes and 39% of ACIOL eyes (*p* = 0.01). **Conclusions:** The iris fixation of late dislocated IOL–capsular bag complexes is a safe and minimally invasive technique that offers better visual outcomes, less astigmatism and fewer complications than ACIOL exchange.

## 1. Introduction

Cataract surgery with the implantation of an intraocular lens (IOL) is a highly successful surgical procedure that restores patients’ vision and quality of life [1]. Sutureless incisions, a continuous curvilinear capsulorhexis, phacoemulsification and the placement of a foldable IOL into the capsular bag are now the gold standard for routine cataract surgery, with the number of operations increasing each year [2]. In addition, improvement of surgical techniques and the development of new tools, such as capsular support devices and iris and capsular hooks, together with intraocular sophisticated microsurgical instruments, offer possibilities for the safe placement of an IOL in the capsular bag, even in difficult cases [3,4,5,6]. Unfortunately, there are a number of known complications following uncomplicated cataract surgery and the spontaneous dislocation of the IOL–capsular bag complex is one of them.

In a recent systematic review, Maximilian et al. identified several risk factors, such as preoperative trauma and zonular dehiscence, previous vitrectomy, retinitis pigmentosa, pseudoexfoliative syndrome, high myopia, glaucoma or previous glaucoma surgery, corneal endothelial damage (often an indication of complicated cataract surgery) and uveitis. In addition, hydrophilic, quadripod and haptic angulation IOLs were mentioned as potential risk factors. Notably, about a quarter of patients presented with no identifiable risk factors [7]. An increasing trend in the incidence of IOL–capsular bag complex dislocation has been reported [8]. This could possibly reflect a true increased incidence, or it could rather be the result of more cases due to a growing pseudophakic population [8].

The literature reports an annual incidence of IOL dislocation after cataract surgery of 0.0–0.032%, and the overall incidence over 10–25 years is 0.1–3% [1,9,10,11,12,13,14,15,16]. However, the exact number of late spontaneous IOL–capsular bag complex dislocations is not known, as most studies have included all types of dislocations, including cases of early or even immediate dislocations at the time of cataract surgery [17]. In addition, the prevalence of IOL dislocations varies by region and other factors, such as pseudoexfoliative syndrome [18]. A recent nationwide population-based study in South Korea by Lee et al. found that the overall probability of IOL dislocation after cataract surgery was approximately 2.73% [17]. However, it is emphasized that pseudoexfoliation syndrome, as a well-known risk factor for IOL dislocation, is lower in Asia than in Western countries [17]. It is possible that the incidence is higher in Europe, as shown by a prospective cohort study in Sweden, which found a 20-year cumulative incidence of 3% and 6% in patients with pseudoexfoliation syndrome [19]. In Lithuania, as in other European countries, a high incidence of pseudoexfoliation syndrome is observed [20]. Therefore, in parallel, the problem of IOL dislocation is also of great importance.

IOL–capsular bag complex dislocation presents significant surgical management challenges and questions for ophthalmologists when it comes to surgical treatment. The two main options are the removal of the IOL and replacement with a new IOL, or the repositioning and fixation of the same IOL in the eye. One of the advantages of IOL repositioning and fixation is that extensive surgical maneuvers, including large incisions and vitreous surgery, can be avoided. This may lead to less corneal endothelial damage and lower postoperative astigmatism [21]. Moreover, replacement of the IOL–capsular bag complex with an anterior or posterior chamber IOL with additional fixation is challenging and carries the risk of complications such as the development of choroidal hemorrhage, macular edema, etc. [21].

It seems that the need to preserve the IOL is becoming more common according to the literature [22,23,24,25,26]. Consequently, much attention is being paid to the development of surgical techniques to expand the options in different cases, especially for anterior segment surgeons [22,23,24,25,26].

The current study focuses on the outcomes of the iris suture late spontaneously dislocated IOL–capsular bag complex and compares it with IOL exchange in a large tertiary reference center in Lithuania.

## 2. Materials and Methods

The prospective observational study was conducted between 2017 and 2019 year at the Department of Ophthalmology, Lithuanian University of Health Sciences, Kaunas, Lithuania. All study procedures were conducted in accordance with the Declaration of Helsinki. Kaunas Regional Ethics Committee approved (12 July 2017) the study protocol (No. BE-2-40) for biomedical research. Written informed consent was obtained from all patients.

The inclusion criterion was a late spontaneous IOL–capsular bag complex dislocation with visible IOL in the pupil area more than 6 months after uncomplicated cataract surgery (by phacoemulsification). IOLs which were completely dislocated into the vitreous where an anterior surgical approach is not possible were not included. Patients who were allergic to local anesthetics or other medications used in ophthalmology; patients with a history of concomitant eye diseases that may have a negative impact on postoperative visual acuity (stage III–IV glaucoma, keratitis, corneal endothelial dystrophy, corneal opacity, pterygium, changes in the macular area of the retina); patients who had had injuries to the eyeball or orbital fractures and deformations, eye surgery (corneal transplantation, pars plana vitrectomy), or laser procedures (refractive laser correction, laser cyclodestruction) in the past; patients with systemic, uncompensated diseases (arterial hypertension, systemic inflammatory diseases, diabetes mellitus type 1 and 2, chronic infectious diseases, conditions after tissue or organ transplantation); patients with one sighted eye (the prognosis for vision in the other eye is poor, the eye is blind or missing); patients with a mental illness; pregnant or breastfeeding women; and patients who did not consent to an examination in the postoperative period were excluded from the study.

A total of 80 patients (83 eyes) with late spontaneous IOL–capsular bag complex dislocation were enrolled. The surgical technique chosen depended on the extent of the IOL dislocation and the patient’s preference to retain their own IOL.

### 2.1. Preoperative Examination

The time interval between cataract surgery and the dislocation of the IOL–capsular bag complex, the IOL type, the presence or absence of a capsular tension ring (CTR) and concomitant ocular diseases were recorded before IOL–capsular bag complex dislocation surgery. In addition, previous glaucoma diagnoses, filtering surgeries and glaucoma medication requirements were recorded, indicating the number of different glaucoma medication drops the patient was taking during the preoperative visit.

Refractive status was examined using autokeratometry (Tonoref III, Nidek, Japan), and spherical and cylindrical powers were converted to the spherical equivalent (SE) (sphere plus half cylinder). Other investigations were conducted, including the following: examination of best corrected distance visual acuity (BCDVA) using the Early Treatment Diabetic Retinopathy Study visual acuity chart from a distance of 5 m; applanation tonometry, using a Goldmann tonometer, slit lamp biomicroscopy and ophthalmoscopy; slit lamp photography of the anterior segment of the eye; and measurement of the corneal endothelial cell density (ECD) with confocal microscopy of the cornea in vivo (Heidelberg Retina Tomography with III Rostock Cornea Module; Heidelberg Engineering GmbH, Heidelberg, Berlin, Germany).

#### The Grade of IOL–Capsular Bag Complex Dislocation

The degree of IOL–capsular bag complex dislocation was evaluated by slit-lamp examination and a photograph of the anterior segment was taken. The dislocation was classified as follows:

- Grade 1: Pseudophacodonesis.
- Grade 2: Small decentration. The IOL is slightly decentered, but the equator of the IOL optic is located behind the iris and outside the pupillary zone. With the narrow pupil, only the decentration of the capsulorhexis (no IOL equator) can be observed.
- Grade 3: Moderate dislocation. The equator of the IOL optic is above or coincides with the line drawn through the center of the pupil.
- Grade 4: Advanced dislocation. The IOL is more dislocated than grade 3 and the equator of the IOL optic is below the line horizontally drawn through the center of the pupil.

The assessment of the degree of dislocation methodology is described in more detail by our group [27].

### 2.2. Surgical Procedure

#### 2.2.1. IOL Repositioning and Fixation to the Iris Procedure (IF Group)

Two paracenteses in the clear cornea are performed along the anticipated path of the needle’s entry and exit. The 10-0 Polypropylene suture with a long curved needle (Alcon Laboratories, Inc., Fort Worth, TX, USA) was used. The needle is passed through the first stab incision and the pupil beneath the haptics of the IOL and guided into the anterior chamber through the iris in a matched place for IOL fixation. The IOL–capsular bag complex can be temporarily stabilized by planting a spatula behind it to facilitate the puncture of a fibrosed capsular bag and to prevent subsequent additional damaging of the residual Zinn’s zonules. A 27-gauge cannula is introduced through the distal paracentesis, and the needle is docked to facilitate exit. The needle is passed back into the eye through the second stab incision, the iris (close to the first perforation), above the IOL haptics, through the pupil, into the anterior chamber and out of the eye through the first stab incision. At this moment, the IOL haptic is encircled by the suture. The first double throw is performed outside the eye. Both suture ends are pulled to make the first floor of the knot behind the iris. The long end of the thread is pulled outside the eye through the second stab incision and the double throw is pulled inside of the eye. A gently tied knot is formed inside the eye and beneath the iris. The long suture is repeatedly drawn through the first stab incision with a microhook, and a second single throw is performed outside the eye. The third single throw is performed in the same manner. The ends of the suture are pulled away, bringing the third throw snugly into position without twisting. The ends of the suture are cut off with vitrectomy scissors or a knife inside the eye and the knot is buried behind the iris. Using this fixation method the pupil remains circular after the procedure. This modified Siepser knot methodology was described earlier [28].

#### 2.2.2. Replacing the IOL–Capsular Bag Complex with Anterior Chamber Intraocular Lens Implant Procedure (ACIOL Group)

Three sclerotomies are performed by 25 G trocars and two paracenteses by a 20 G knife. An infusion line is inserted into the trocar and after checking its full penetration through pars plana balanced saline solution (BSS), infusion is started. After the full pars plana vitrectomy is performed, an iridotomy at 12 o’clock is made by the vitrector at a slow cutting rate.

The main 6 mm corneal incision is made by a knife. The old IOL is removed and a new IOL (MTA4U0 (Alcon Laboratories, Inc., Fort Worth, TX, USA)) is implanted into the anterior chamber of the eye. The IOL is rotated into a horizontal position. The main corneal incision is sutured with three single-knotted 10/0 Ethylon sutures. The paracenteses are closed by injecting a balanced salt solution into the corneal stroma. The trocars are removed from the sclerotomies.

### 2.3. Postoperative Examination

All patients underwent corneal suture removal 3 months after replacing the IOL–capsular bag complex with the anterior chamber lens surgery. Six months after the surgery, all participants underwent the same ophthalmic examination as before surgery. Postoperative complications were recorded. After surgery, to evaluate the status of the macular area, optical coherence tomography (DRI OCT Triton plus (Ver.10.13), Topcon, Japan) was performed. We investigated whether cystoid macular edema (CME) was present and measured the postoperative central macular thickness.

### 2.4. Statistical Analysis

Statistical analysis was performed using IBM SPSS Statistics for Windows, Version 29.0.2.0, Armonk, NY, USA: IBM Corp. The Kolmogorov–Smirnov test was used to assess the distribution of quantitative data. For quantitative variables that did not meet the assumption of normality, the differences between groups were evaluated using non-parametric tests: the Mann–Whitney U test for comparisons between two independent groups, the Kruskal–Wallis test for comparisons among more than two independent groups and the Wilcoxon signed-rank test for comparisons between two dependent groups. For normally distributed variables, comparisons were performed using Student’s *t*-test—specifically, the independent samples *t*-test for two independent groups and the paired *t*-test for two dependent groups. The association between two qualitative variables was assessed using the Chi-square (χ^2^) test. The data are presented as the mean (standard deviation (SD)), minimum (min.) and maximum (max.) values, median (interquartile range (IQR)) or number (percentage), and *p* < 0.05 is considered statistically significant.

## 3. Results

### 3.1. Demographic Data

In total, 80 patients (83 eyes) (30 female, 50 male) were included in the study. A total of 43 eyes were in the IF group, and 40 eyes were in the ACIOL group. The mean age of the patients at the moment of diagnosed IOL dislocation was 77.08 ± 7.28 years. The mean age at the moment of cataract surgery was 69.45 ± 8.02 years. The mean time from cataract surgery to IOL dislocation was 7.64 ± 3.88 years. Baseline characteristics and preoperative study parameters are presented in Table 1.

Age, gender and time since cataract surgery did not differ between the two groups. Also, the data related to the cataract surgery (IOL type, CTR presence and previous laser capsulotomy) did not differ between the groups. Trabeculectomy (TB) prior to IOL dislocation was performed more frequently in the IF group than in the ACIOL group. However, this difference was not significant (Table 1).

### 3.2. Visual Outcomes

**Figure 1:**
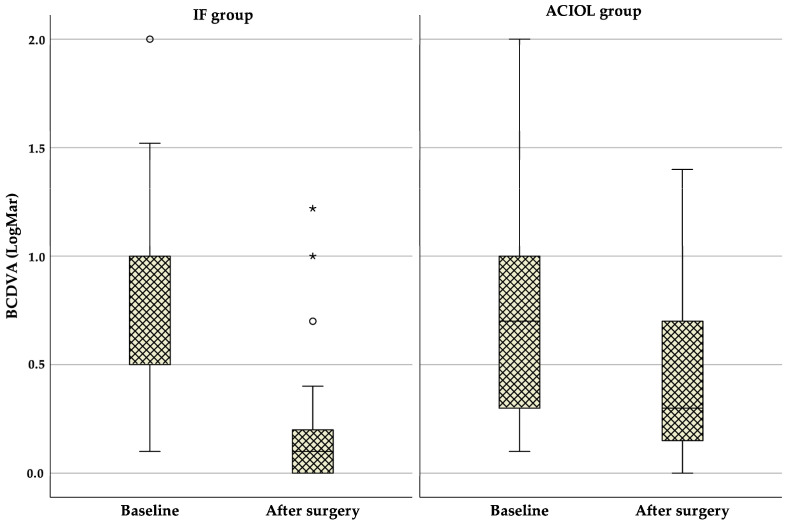
Boxplot of BCDVA changes in the two groups before and after surgery. ACIOL = anterior chamber intraocular lens, BCDVA = best corrected distance visual acuity, IF = iris fixation. Circles indicate mild outliers (1.5 × IQR), while stars represent extreme outliers (3 × IQR).

As shown in Figure 1, median BCDVA statistically significantly improved throughout the follow-up period in both groups, increasing from 0.5 (1.0–0.5) logMAR at baseline to 0.1 (0.2–0.0) logMAR at 6 months in the IF group (Z = −5.13, *p* < 0.001), and from 0.7 (1.0–0.3) logMAR at baseline to 0.3 (0.7–0.13) logMAR at 6 months in the ACIOL group (Z = −3.223, *p* = 0.001). No statistically significant differences were observed between the two groups at baseline BCDVA (*p* = 0.508), but it was significantly better in the IF group after 6 months (*p* = 0.001).

During the follow-up period, there was no statistically significant change in corneal astigmatism in the IF group’s (median cylinder 1.10 (0.65–1.55) D at baseline and 0.86 (0.41–1.39) D at 6 months, (Z = −1.863, *p* = 0.62). However, the change was significant in the ACIOL group’s 6 months after surgery (median cylinder 1.01 (0.53–1.51)) D at baseline and 2.8 (1.51–4.72) D at 6 months, (Z = −4.888, *p* = 0.001). No significant differences in corneal astigmatism were found between the two groups at baseline (*p* = 0.613), but after 6 months, corneal astigmatism was significantly higher in the ACIOL group (*p* < 0.001).

The median SE changed statistically significantly from 1.38 (−0.63–6.38) D at baseline to −0.75 (−1.63–−0.13) D at 6 months (Z = −3.797, *p* < 0.001) in the IF group and from 5.38 (0.47–10.63) D at baseline to −1.31 (−1.97–−0.63) D at 6 months (Z = −5.498, *p* < 0.001) in the ACIOL group. The SE was significantly higher in the ACIOL group at baseline (*p* = 0.002) and did not differ significantly between the groups after 6 months (*p* = 0.001).

### 3.3. IOP and Glaucoma Treatment Outcomes

A total of 45 (54.22%) eyes were diagnosed with primary open angle glaucoma before the IOL–capsular bag complex dislocation. A total of 15 (18.1%) eyes were diagnosed with secondary glaucoma at the time of the IOL–capsular bag complex dislocation. Taken together, this represents 60 (72.32%) eyes of the study participants. The most commonly used preoperative eye drops were beta-blockers (44 (55.4%) eyes) and carbonic anhydrase inhibitors (31 (37.3%) eyes) (prostaglandins—29 (34.9%) eyes, alpha-mimetics—11 (13.3%) eyes and prostamides—9 (10.8%) eyes). Systemic carbonic anhydrase inhibitors were administered in six (7.2%) patients. All investigated eyes (n = 83) had a median preoperative IOP of 19.0 (16.0–22.0) mmHg. Despite medical treatment, an IOP ≥ 21 mmHg before surgery for IOL dislocation was found in 24 (28.9%) eyes, and half of them (12 (50%) eyes) even had an IOP ≥ 30 mmHg.

**Figure 2:**
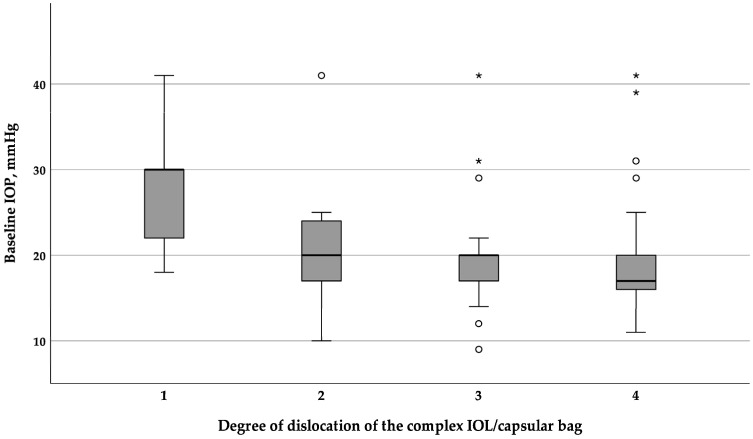
Boxplot of the preoperative IOP drop with the increasing degree of IOL–capsular bag dislocation. IOL = intraocular lens, IOP = intraocular pressure. Circles indicate mild outliers (1.5 × IQR), while stars represent extreme outliers (3 × IQR).

The analysis of the preoperative IOP in relation to the grade of IOL dislocation revealed a statistically significantly higher IOP with a grade 1 dislocation compared to grades 2, 3 and 4 (χ^2^ = 13.783, df = 3, *p* = 0.003) (Figure 2).

**Figure 3:**
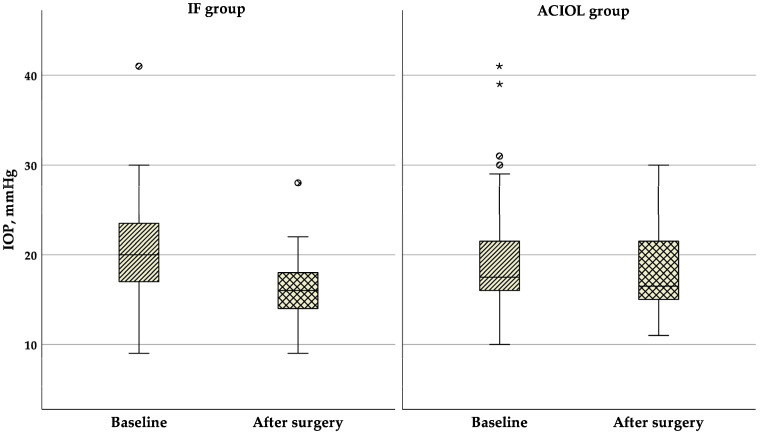
Boxplot of IOP changes in the two groups before and after surgery. ACIOL = anterior chamber intraocular lens, IF = iris fixation, IOP = intraocular pressure. Circles indicate mild outliers (1.5 × IQR), while stars represent extreme outliers (3 × IQR).

As shown in Figure 3, IOP decreased throughout the follow-up period in both groups, decreasing from 20.0 (17.0–25.0) mmHg at baseline to 16.0 (14.0–18.0) mmHg at 6 months in the IF group (Z = −4.710, *p* < 0.01), and from 17.5 (16.0–21.75) mmHg at baseline to 16.5 (15.0–21.75) mmHg at 6 months in the ACIOL group (Z = −0.684, *p* = 0.494). There was no significant difference in IOP between the groups before or after IOL dislocation surgery (*p* = 0.248 and *p* = 0.057, respectively), but the change was statistically significantly bigger in the IF group (*p* = 0.004).

The changes in the IOP-lowering medical treatment between the preoperative and postoperative visits are shown in Table 2.

The amount of antiglaucoma medication did not change statistically significantly in either group when comparing the results before and after IOL dislocation treatment (IF group *p* = 0.519, ACIOL group *p* = 0.159). In the IF group, the number of antiglaucoma drops did not change in twenty-two (70.97%) eyes, increased in four (12.90%) eyes and decreased in five (16.13%) eyes. In the IF group, three of twelve eyes that were not diagnosed with glaucoma before IOL dislocation treatment started with antiglaucoma therapy after surgical IOL dislocation treatment. In the ACIOL group, the amount of antiglaucoma drops did not change in eighteen (62.07%) eyes, increased in eight (27.59%) eyes and decreased in three (10.34%) eyes.

Four (9.3%) eyes from the IF group and one (2.5%) eye from the ACIOL group underwent filtering surgery for subcompensated IOP during the 6-month period after IOL dislocation treatment (*p* = 0.193). In the IF group, the mean IOP of these eyes was 38.0 mmHg before IOL dislocation treatment and 11.5 mmHg 6 months after IOL dislocation treatment and filtering surgery, without antiglaucoma medication. In the ACIOL group, the IOP of the eye with additional filtering surgery was 31.0 mmHg before IOL dislocation and 15.0 mmHg 6 months after IOL dislocation treatment and filtering surgery. Three drops of an antiglaucoma medication were administered daily to support the IOP.

### 3.4. Other Postoperative Outcomes

**Figure 4:**
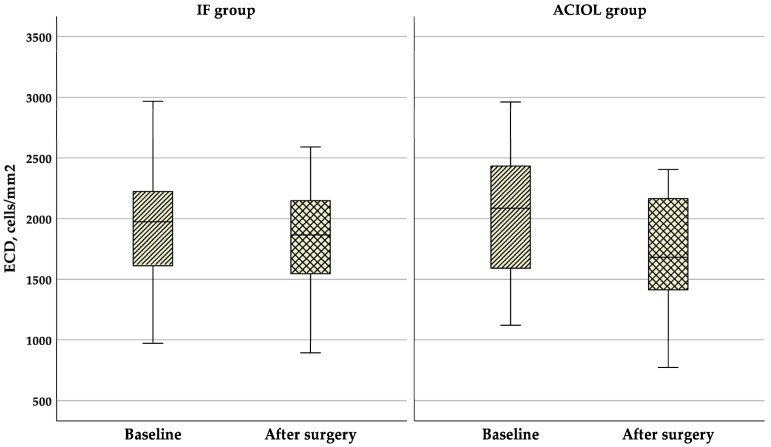
Boxplot of ECD changes in the two groups before and after surgery. ACIOL = anterior chamber intraocular lens, ECD = endothelial cell density, IF = iris fixation.

As shown in Figure 4, mean ECD significantly decreased throughout the follow-up period in both groups, from 1922 ± 468 cells/mm^2^ at baseline to 1800 ± 433 cells/mm^2^ at 6 months in the IF group (*p* < 0.001), and from 2021 ± 480 cells/mm^2^ at baseline to 1683 ± 446 at 6 months in the ACIOL group (*p* < 0.001). There was no significant difference in ECD between the groups before or after IOL dislocation surgery (*p* = 0.347 and *p* = 0.230, respectively), but the change was statistically significantly bigger in the ACIOL group (*p* < 0.001).

CME was found in 18 of 83 eyes (21.69%) after surgery. Statistically significantly less cases occurred in the IF group than in the ACIOL group (two (4.4%) vs. sixteen (39%), respectively, *p* = 0.01). The central macular thickness was higher in the ACIOL group (*p* = 0.01) (Table 2). In three (6.7%) of the forty-three eyes in the IF group, the IOL was re-fixated due to the insufficient centering of the IOL. An improvement was observed in these patients after topical non-steroidal anti-inflammatory drugs were administered.

There was no intraocular hemorrhage, fluid misdirection syndrome during surgery, bullous keratopathy, anterior pigmentary dispersion syndrome, chronic uveitis, retinal detachment or endophthalmitis during the 6-month period after surgery in both groups.

## 4. Discussion

Over the decades, a number of surgical techniques have been developed to treat IOL dislocations. These include removal of the dislocated IOL and secondary IOL implantation (anterior chamber IOL, iris-claw IOL, iris-fixated IOL, scleral-fixated IOL with suture, scleral-fixated IOL without suture) or repositioning and fixation of the same IOL to the sclera or iris, each with its benefits and risks [29,30,31,32,33]. Despite numerous studies on this topic, however, no single technique has gained an advantage over the others [30,31,32,33,34,35]. Nevertheless, complications such as corneal decompensation, glaucoma, chronic inflammation and CME are more common with IOL exchange (due to the large incision wounds and longer operating times) than with other treatment options, including the repositioning of a dislocated IOL using a scleral or iris fixation in the globe as a closed system [36]. The scleral fixation of IOLs, especially the sutureless technique, reduces the risk of many of these complications [32]. However, it is considered the most technically challenging technique requiring a posterior segment surgeon [32]. When performing this to a deep scleral groove, a microvitreoretinal knife or needle can traumatize the ciliary vessels, which carries the risk of suprachoroidal hemorrhage (incidence after sutured scleral fixation is up to 3% and rarely occurs after sutureless scleral fixation) [37]. On the other hand, extrusion of the haptics (slippage of the haptics from the scleral tunnel) may occur due to thin scleral flaps, poor scleral tunnel construction or adhesion, excessive use of scleral cauterization and congenital or acquired fragility of the sclera [38]. Reported rates vary from 0.8% to 12.5% [38]. Permanent externalized sutures or the ends of cauterized haptics may also increase the risk of late endophthalmitis [39,40]. Another common problem with sutureless fixation is the instability of the IOL [41]. Also, the increased iris flutter caused by eye movement can lead to a reverse pupillary block due to the absence of the barrier behind the iris, which prevents the aqueous humor and vitreous humor from flowing into the anterior chamber [42].

To expand the options for anterior segment surgeons, we have focused on the technique of the iris fixation for a dislocated IOL–capsular bag complex. The technique we have presented is safe and minimally invasive. When performing IOL fixation with this technique, there are no blind movements of the needle behind the iris. The haptics (with the surrounding capsule) are sutured to the iris under direct observation under the microscope, precisely in the anticipated location with no or minimal pupil deformation, with the knot tied behind the iris. Fixation of the dislocated IOL to the iris using McCannel sutures is generally performed in a ‘blind’ manner (when the needle is behind the iris) [43]. This may cause the improper localization of the haptic and suturing to the iris in the unintended place. Furthermore, it is challenging to penetrate the iris from behind close to the first prick. In addition, the presence of a fibrous capsular bag increases the risk due to IOL luxation into the vitreous. The described surgical technique of the additional stabilization of the IOL–capsular bag complex with an instrument (spatula) during surgery prevents damage to the remaining Zinn’s zonules. In addition, another unique feature is that the Siepser slipknot remains behind the iris (between the posterior surface of the iris and the IOL–capsular bag complex). We believe that creating this small space between these structures has positive properties. After such fixation, none of the patients had pigment dispersion syndrome or uveitis–glaucoma–hyphema syndrome. This technique also does not require large incisions in the cornea or sclera and does not harm the conjunctiva [24]. As 72.3% of patients had concomitant glaucoma, it was important to find the least traumatic method of repositioning the IOL to spare the superior conjunctiva and sclera in patients if subsequent filtration surgery should be required due to glaucoma. This technique can be used by anterior segment surgeons as no additional surgical intervention is required. However, experience with McCannel suturing and tying a Siepser slipknot is essential to maintain the minimally invasive nature of this technique.

Our prospective study is unique because we have included cases of late spontaneous IOL–capsular bag complex dislocation treated with the repositioning of the same IOL using iris suture fixation or IOL exchange into the anterior chamber IOL. Early in our study, the IOL exchange into the anterior chamber IOL was preferred. Still, the trend shifted from IOL exchange to iris-claw IOL or IOL repositioning as these techniques developed and improved.

In the IF group, very encouraging visual and refractive results were achieved. This supports other authors’ findings from studies using the iris suturing of modified McCannel sutures in cases with IOLs without capsular support or in cases with IOL–capsular bag complex dislocation [43,44]. In most patients in the IF group (with the exception of three patients (6.7%) who required re-fixation due to inadequate IOL centration), we observed an improvement in BCDVA. Significant improvement in visual acuity was also observed in the ACIOL group. However, it was significantly less than in the IF group due to the significant increase in corneal astigmatism. Often, anterior chamber or iris-claw IOLs are implanted, but large corneal incisions (approximately 6 mm) are required, which can increase corneal astigmatism. Our technique of IOL repositioning with iris fixation does not require a large corneal incision, and this is the reason for minor changes in the corneal cylinder. Condon et al. included forty-six patients who underwent foldable acrylic IOL implantation using peripheral iris suture fixation for aphakia without capsule support. They also observed no significant changes in corneal astigmatism [43]. The results of the study by Michaeli et al. also confirm these findings [45]. Furthermore, a more anterior position of the lens caused a myopic shift, which was acceptable for the patients. In a retrospective study, Faria et al. examined 36 eyes with dislocated IOLs treated by an iris suture and also found a myopic shift in all patients [46]. Our study data also support this.

Patients with IOL dislocation often have increased IOP [47,48,49,50]. Anatomical changes in the anterior segment of the eye, irritation of the ciliary body and possibly inflammatory processes could cause an increase in IOP. It is possible that all these factors are less pronounced with a higher degree of IOL–capsular bag complex dislocation. This could explain why IOP decreases with an increasing degree of IOL dislocation. In fact, Bulnes et al. found the same significant association between IOP and the degree of IOL dislocation [47]. This aspect needs further and more detailed investigation.

IOP and the need for glaucoma medication did not change statistically significantly in either of our study groups. However, using anterior chamber IOL implants may be associated with IOP elevation and glaucoma development, whereas eyes with iris-sutured IOLs were less prone to IOP increase. In our series, we found fifteen patients (18.07%) with postoperative IOP elevation that was resolved with topical therapy (seven in the IF group and eight in the ACIOL group). In five patients with high preoperative IOP (>30 mmHg), filtering surgery was performed within 6 months after treatment of IOL dislocation to control IOP. We cannot extrapolate from a few cases to others, but our technique of IOL repositioning with iris fixation does not harm the sclera and conjunctiva as we expected. In four cases, the target IOP was achieved after IOL repositioning with iris fixation and filtering surgery without medication. After IOL exchange and filtering surgery in one case, three drops of antiglaucoma medication were administered daily to support the target IOP.

A severe reduction in ECD after intraocular surgery can lead to serious complications such as bullous keratopathy; therefore, minimally invasive surgery is always preferred in this regard [51]. In our study, we observed a smaller decrease in ECD in the IF group. Dzhaber et al. retrospectively examined 117 eyes in which iris fixation was used and recorded a decrease in ECD of almost 200 cells/mm^2^ [52]. Kim et al. recorded a similar decrease (12.7% ± 8.7%) in ECD after iris fixation, and this change was not different from the transscleral fixation group (10.9% ± 9.2%) (*p* = 0.16) [53].

In our study, we found that CME occurred more frequently in the ACIOL group. Anatomically, the close proximity of the haptics of the ACIOL to the iridocorneal angle can cause chronic inflammation and, consequently, CME [44]. As found in previous studies, the rate of CME in iris-fixated IOLs is equal to or lower than in scleral-fixated and anterior chamber IOLs [54]. A randomized trial comparing the three IOL fixation strategies in 176 patients lacking adequate capsule support has been published [55]. Although the visual outcomes were similar in the three groups, it was found that iris-sutured IOLs were associated with significantly less CME (20%) versus anterior chamber IOLs (38%) or scleral-sutured IOLs (41%) (*p* = 0.02) [55]. Our study results do not contradict these findings.

In a retrospective study, Caporossi et al. reported on 41 eyes with IOL dislocation treated with the repositioning of the same IOL using iris suture fixation in two groups (acrylic and rigid one-piece IOL group and acrylic three-piece IOL group) [25]. They achieved good refractive results, a stable IOL, no increase in surgically induced astigmatism and a low number of complications (no intraoperative complications, endothelial dysfunction and pigment dispersion). The results did not differ between groups. In summary, they concluded that the iris fixation technique for one- and three-piece IOLs is a safe and valid option for treating IOL dislocations. Our results do not contradict these findings.

In the literature review, we found no methods for a sutureless/automated fixation technique of subluxated IOLs to the iris, which could be the aim of further studies. Our study has several limitations, including bias due to patients’ preference to retain their own dislocated IOL, a relatively short postoperative follow-up period and a single-center study design. Therefore, a randomized multicenter trial with a longer follow-up is warranted.

## 5. Conclusions

Our study findings indicate that iris fixation is a safe and effective technique for managing dislocated IOL–capsular bag complexes, offering a favorable profile of minimal complications and good morphological and functional outcomes. A key advantage is the ability to reuse the same IOL within a closed eye, thus avoiding a large incision. Nonetheless, further prospective studies with longer follow-up and multicenter collaborations are warranted to validate these results. Moreover, the development of sutureless or automated iris fixation techniques remains an important goal for future research, as it could streamline the surgical approach and potentially reduce complications even further.

## Figures and Tables

**Table 1:** Late spontaneous IOL–capsular bag complex dislocation: baseline characteristics.

	IF Group N of Eyes = 43	ACIOL Group N of Eyes = 40	*p*
	Mean (SD) (min.–max.)		
Age (years)	76.91 (8.08) (48–98)	77.28 (6.40) (60–92)	0.820 *
Age at the cataract surgery (years)	68.44 (8.9) (29–82)	70.53 (6.9) (58–90)	0.239 *
Time since cataract surgery (years)	8.26 (4.1) (3–19)	6.98 (3.61) (1–14)	0.134 *
	N (%)		
Gender (male/female)	25 (60.98)/16 (39.02)	22 (56.41)/17 (43.59)	0.623 **
IOL material:			
Hydrophobic acrylic	29 (67.4)	24 (60)	0.481 **
Hydrophilic acrylic	14 (32.6)	16 (40)	
CTR presence	12 (27.9)	22 (45)	0.105 **
Laser capsulotomy	3 (7.0)	5 (10.0)	0.620 **
IOL dislocation grade:			
1	8 (17.8) ^a^	1 (2.4) ^a^	**^a^** 0.016 **
2	8 (20.0)	7 (17.1)	
3	14 (33.3)	8 (22.0)	
4	13 (28.9) ^a^	24 (58.5) ^a^	
TB	8 (18.6)	2 (5.0)	0.057 **

ACIOL = anterior chamber intraocular lens, CTR = capsular tension ring, IF = iris fixation, IOL = intraocular lens, N = number, TB = trabeculectomy; ^a^ denotes the difference between groups, * *t*-test, ** χ^2^ test.

**Table 2:** Outcome after surgery for late spontaneous IOL–capsular bag complex dislocation in the two surgical groups.

	IF Group N of Eyes = 43	ACIOL Group N of Eyes = 40	*p*
	Median (IQR)		
N of IOP-lowering medication, drops	2 (0–3)	2 (0–3)	0.272 *
N of IOP-lowering medication change, drops	0 (0–0)	0 (0–0.75)	0.119 *
Macular thickness, µm	262.6 (252.65–271.63)	303.7 (269.92–338.23)	0.01 *
	N (%)		
CME	2 (4.4)	16 (39)	0.01 **
TB	4 (8.9)	1 (2.4)	0.363 **
Refixation	3 (6.7)	0	0.243 **

ACIOL = anterior chamber intraocular lens, CME = cystoid macula edema, IF = iris fixation, N = number, TB = trabeculectomy; * Mann–Whitney test, ** χ^2^ test.

## Data Availability

The datasets used and/or analyzed during the current study are available from the corresponding author on reasonable request.
